# Prenatal Smoking Might Not Cause Attention-Deficit/Hyperactivity Disorder: Evidence from a Novel Design

**DOI:** 10.1016/j.biopsych.2009.05.032

**Published:** 2009-10-15

**Authors:** Anita Thapar, Frances Rice, Dale Hay, Jacky Boivin, Kate Langley, Marianne van den Bree, Michael Rutter, Gordon Harold

**Affiliations:** a Department of Psychological Medicine, School of Medicine, Cardiff University, Cardiff, Wales; b School of Psychology, Cardiff University, Cardiff, Wales; c Institute of Psychiatry, King's College London, United Kingdom

**Keywords:** ADHD, genetic, maternal smoking, maternal smoking in pregnancy, prenatal

## Abstract

**Background:**

It is widely considered that exposure to maternal cigarette smoking in pregnancy has risk effects on offspring attention-deficit/hyperactivity disorder (ADHD). This view is supported by consistent observations of association. It is, however, impossible to be certain of adequate control for confounding factors with observational designs. We use a novel “natural experiment” design that separates prenatal environmental from alternative inherited effects.

**Methods:**

The design is based on offspring conceived with Assisted Reproductive Technologies recruited from 20 fertility clinics in the United Kingdom and United States who were: 1) genetically unrelated, and 2) related to the woman who underwent the pregnancy. If maternal smoking in pregnancy has true risk effects, association will be observed with ADHD regardless of whether mother and offspring are related or unrelated. Data were obtained from 815 families of children ages 4 years–11 years with parent questionnaires and antenatal records. Birth weight was used as a comparison outcome. The key outcome considered was child ADHD symptoms.

**Results:**

Association between smoking in pregnancy and lower birth weight was found in unrelated and related mother-offspring pairs, consistent with a true risk effect. However, for ADHD symptoms, the magnitude of association was significantly higher in the related pairs (β = .102, *p* < .02) than in the unrelated pairs (β= −.052, *p* > .10), suggesting inherited effects.

**Conclusions:**

Our findings highlight the need to test causal hypotheses with genetically sensitive designs. Inherited confounds are not necessarily removed by statistical controls. The previously observed association between maternal smoking in pregnancy and ADHD might represent an inherited effect.

It has generally come to be accepted that exposure to maternal smoking in pregnancy has a causal role in offspring attention-deficit/hyperactivity disorder (ADHD). It is biologically plausible, because it is known that smoking has an effect on physiological processes that might create risks relevant to the origins of ADHD, a childhood-onset neurodevelopmental disorder (1,2). Confidence that the causal inference is correct comes from a combination of the fact that associations have been consistent across a range of studies and that the relationship has held up after statistically controlling for possible confounding factors (3,4). It is difficult, however, to provide adequate control for confounding factors in observational studies. Randomized controlled trials provide a powerful experimental means of testing causal hypotheses, but randomization of exposure for maternal smoking in pregnancy is neither feasible nor ethical in humans. Therefore, “natural experiments” that pull apart the effects of prenatal risk factors from alternative mediating mechanisms are needed (5–7).

A particular concern is that the relationship between maternal smoking in pregnancy and offspring ADHD arises from unmeasured confounds, including inherited ones. Fetal exposure to prenatal risk factors, especially those linked to offspring mental health, is not random (8). Public health initiatives emphasize the importance of prospective mothers quitting smoking. Nonetheless, a significant proportion of mothers continue to smoke during pregnancy. This behavior is influenced by a range of maternal characteristics, including maternal genetic factors (9). As mothers transmit genes to their offspring, the link between maternal smoking and offspring outcomes could be attributable to inherited effects, in addition to any true environmentally mediated effect (e.g., the effects of cigarette toxins). We have employed a novel design that allows us to remove the inherited link between offspring and mothers and test prenatal risk effects alone. The unique design is based on investigating the offspring of women who were conceived through assisted reproduction technologies (ART), because it becomes possible to examine children who are genetically unrelated to the woman who undergoes the pregnancy (10). If the maternally provided prenatal environmental risk factor has true risk effects on outcome, then associations will be observed regardless of whether the woman who underwent the pregnancy is related or unrelated to her child. In contrast, if observed only in those that are related, this indicates that the association between prenatal risk factor and outcome arises by virtue of maternally provided inherited factors. We employ this design to test claims that maternal smoking in pregnancy has risk effects on offspring ADHD (4). The design was validated by undertaking identical analyses comparing the effects of maternal smoking in pregnancy on birth weight, where effects are known to be causal (11–16).

## Methods and Materials

### Sample

**Figure 1:**
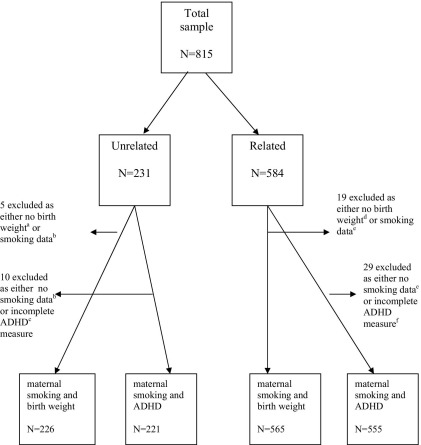
Sample description Unrelated: ^a^no birth weight data alone, *n* = 2; ^b^no smoking data alone, *n* = 5; ^c^incomplete attention-deficit/hyperactivity disorder (ADHD) measure alone, *n* = 9. Related: ^d^no birth weight data alone, *n* = 10; ^e^no smoking data alone, *n* = 14; ^f^no ADHD measure alone, *n* = 25. No prenatal data available was due either to mothers not completing questionnaire or to not giving consent to obtain birth records. Incomplete ADHD measure was due either to mothers not completing questionnaire or to missing some items on the measure.

The research protocol was approved by the Wales Multi-Centre Research Ethics Committee. The sample (Figure 1) consisted of 815 families recruited over 5 years from 19 fertility clinics across the United Kingdom and 1 from Boston, Massachusetts. In 231 families, mother–child pairs were genetically unrelated, because of 172 oocyte donations, 36 embryo donations, and 23 offspring of unrelated gestational surrogates (where fertilized eggs from the social mother are implanted into the “surrogate” who undergoes the pregnancy on behalf of the social mother). In 584 families, mothers were genetically related to their offspring, including 395 homologous in vitro fertilization and 189 sperm donations (Table 1).

Families of school-aged children were invited to participate. Children's ages ranged from 4 years to 11 years (mean = 6.7, SD = 1.3). The sample included 416 boys and 397 girls (2 had missing data), 626 singletons and 187 individuals who were the first born of multiple births (168 twins, 19 triplets; 2 missing data). Ninety-six percent of mothers and 95% of fathers were of European origin. Further description on the sample is available (10,17). Overall the sample is comparable to the general population, although mothers were older and there were more multiple births (17).

### Measures

Postal questionnaires were completed by 800 mothers and 552 fathers. Mothers from the United Kingdom were also asked for consent to examine their antenatal records for the index child, and 616 agreed (77%).

### Outcome Variables

#### Children's ADHD Symptoms

Children's ADHD symptoms during the past 3 months were assessed with the 14-item DuPaul ADHD scale, rated on a 4-point scale (0 = not at all, 1 = just a little, 2 = pretty much, 3 = very much). The summed score for each of the items provides total ADHD scores (18,19). Because most studies have relied on maternal reports of child ADHD, this was the key outcome measure. Total child ADHD scores ranged from 0 to 42 (mean = 10.6, SD = 7.5).

#### Birth Weight

Mothers also reported on this “comparison” outcome. For birth weight, there was excellent agreement between their retrospective reports and antenatal records (20), and thus to maximize sample size, we used maternal reports of birth weight. Mean birth weight was 3070 g (range 595–4989, SD = 700).

### Predictor Variable

Smoking in pregnancy was assessed by a maternally rated questionnaire (19) that has excellent agreement with antenatal records (20). Mothers were considered to have smoked during pregnancy if this was endorsed in their own questionnaire or in their antenatal records. Seven hundred forty-eight mothers did not smoke during pregnancy, and 48 (6%) smoked (no data on 19).

### Measures Used in Additional Analyses

#### Alcohol Use in Pregnancy

Alcohol use in pregnancy (yes/no) was assessed by maternal questionnaire alone, because we found this information was not reliably recorded in antenatal records (20). One hundred eighty-nine mothers (24%) used alcohol during pregnancy.

#### Prematurity

Mothers reported in which week of pregnancy the child was born. The correlation between maternal report and information from antenatal records approached unity (*r* = .959, *p* = .001). Premature birth (*n* = 91, 12.5%) was defined as <37 weeks gestation for singletons and <34 weeks for multiple births.

#### Current Maternal Smoking and Paternal Smoking in Pregnancy

Ninety mothers reported they currently smoked (11.6%). Fathers were also asked whether they smoked during the mother's pregnancy (yes = 115, 21%).

#### Current Annual Family Income

Current annual family income was also assessed by maternal questionnaire. The mean income band was £30–40,000, range <£10,000 to >£60,000.

#### Parent ADHD Symptoms

Each parent was also asked to report on his or her own current ADHD symptoms during the past 3 months with the 18-item Barkley Adult ADHD assessment (21), rated on a 4-point scale (0 = never or rarely, 1 = sometimes, 2 = often, 3 = very often) and summed to yield a total ADHD score. Mother ADHD scores (*n* = 777) ranged from 0 to 34, mean score 8.4 (SD = 5.9). Father ADHD scores (*n* = 529) ranged from 0 to 45, mean score of 8.3 (SD = 6.3).

### Statistical Analysis

#### Primary Analyses

Association between maternal smoking in pregnancy and offspring ADHD scores in the related mother–child pairs and the unrelated mother–child pairs was examined with ordinary least squares regression analysis, where β is the standardized regression coefficient (a measure of the magnitude of association) and the *p* value indicates the statistical significance of that association. The difference in the magnitude of association between smoking in pregnancy and ADHD in the related and unrelated pairs was tested by undertaking multiple regression analysis with the whole sample and including a product term. Here the independent variables were maternal smoking in pregnancy and the product term (smoking × genetic relatedness). The dependent variable was ADHD scores.

Results are also presented for the comparison outcome measure of birth weight where, in contrast to offspring ADHD, it is known that maternal smoking in pregnancy has prenatal risk effects (11–16). Because items from some measures were not available for some families, the numbers of subjects used in each analysis are shown in Figure 1.

### Explanation of Design

#### Testing for Environmental Pathway-Consistency with Prenatal Risk Effects

Significant association in unrelated mother–child pairs must be due to environmental pathways.

#### Testing for Inherited Pathways

Association in the genetically related pairs and greater magnitude of association between prenatal factors and outcome in related than in unrelated pairs is attributable to inherited pathways.

### Additional Analyses

Where there was evidence for association, we tested whether this was attributable to measured child, parent, and family factors, and repeated analyses including covariates with multiple regression analyses. We selected variables that are associated with ADHD symptoms or that could be associated with ADHD or maternal smoking in pregnancy on the basis of previous literature (2,4). Age, gender of child, multiple birth status, maternal alcohol use, birth weight, gestational age, maternal and paternal ADHD symptoms, and family income were considered as covariates.

As a final test of the design, we tested for association: 1) between current maternal smoking and offspring ADHD scores, and 2) between paternal smoking in pregnancy and offspring ADHD for which we know there are no biological explanations of causal effects. For the analyses on paternal smoking, we examined offspring who were genetically related to father and unrelated to father (regardless of maternal relatedness).

## Results

### Description of Sample

Comparisons of the families where mothers are genetically related and unrelated are shown in Table 2. Mothers in the unrelated group were older, and the children were younger. Mean ADHD scores were also lower in the unrelated group.

### Validity of the Design

In this sample, as previously described (22), we find that association between maternal smoking in pregnancy and lower offspring birth weight is present equally in both related (mean birth weight of smokers vs. nonsmokers = 2587 g vs. 3070 g) and unrelated mother–child pairs (2806 g vs. 3098 g). Thus, our design shows that the effects of smoking in pregnancy on birth weight are attributable to environmental pathways (22) (in the genetically related pairs: β = −.14, *p* < .01; unrelated pairs: β = −.11, *p* < .01; no significant differences in magnitude between the groups, *p* > .1, adjusting for maternal education, maternal height, child gender, multiple birth, and maternal age at birth of child and excluding very preterm births [<31 weeks]).

### Association Between Maternal Smoking in Pregnancy and Offspring ADHD Symptoms

Smoking in pregnancy was—in keeping with observational study findings (where mothers and children are genetically related)—significantly associated with offspring ADHD symptoms in the related mother–child pairs (β = .102, *p* < .02) with a mean ADHD score of 13.1 (SD 8.7, *n* = 37) for those exposed and 10.0 (SD 7.4, *n* = 518) for those unexposed to smoking in pregnancy. Statistically controlling for the effects of alcohol use during pregnancy did not alter the results (smoking: β = .101, *p* < .02; alcohol use: β = .027, *p* > .10). Smoking in pregnancy was not related to maternally rated offspring ADHD scores in the unrelated pairs (β = −.052, *p* > .10). The mean ADHD score was 9.8 (SD 5.7, *n* = 9) for those exposed to maternal smoking in pregnancy and was 11.7 (SD 7.6, *n* = 212) for those unexposed. Testing for differences in the magnitude of association in the unrelated and related groups showed evidence of significant differences between the groups (smoking × genetic relatedness: β = −.10, *p* < .05), suggesting significant inherited effects.

### Additional Analyses

#### Covariates

To test whether the association found in the related group was attributable to measured covariates, we then re-ran analyses for maternal smoking in pregnancy controlling for covariates (Table 3). Because alcohol use in pregnancy, birth weight, and gestational age were not associated with ADHD, they were not further included as covariates. Including covariates did not change results for maternal smoking in pregnancy; the evidence for significant association remained in the related mother–child pairs.

#### Current Maternal Smoking and Paternal Smoking in Pregnancy

Finally we examined, as the predictor variables: 1) current maternal smoking, and 2) paternal smoking in pregnancy. In the genetically related pairs, there was evidence of association between current maternal smoking and offspring ADHD (β = .09, *p* < .04) as well as between paternal smoking in pregnancy and offspring ADHD (for these analyses we use offspring who are genetically related to father; β = .114, *p* < .05). There was no association in the unrelated pairs for current maternal smoking (β = −.016, *p* > .10) and when paternal smoking in pregnancy was examined in unrelated fathers only (i.e., sperm and embryo donation for these analyses; β = .031, *p* > .10).

## Discussion

With a novel natural experiment design, we were able to separate prenatal and inherited effects in offspring of maternal smokers. We found, in line with previous research, smoking in pregnancy to be associated with offspring ADHD symptoms in the related mother-offspring pairs and that the association was not accounted for by measured confounds including parent ADHD. The magnitude of association was significantly greater in the related mother-offspring pairs than in the unrelated pairs, which suggests that the well-established link between maternal smoking in pregnancy and offspring ADHD symptoms might represent an inherited rather than a true environmental risk effect. However, our results illustrate that the link between maternal smoking in pregnancy and the comparison outcome variable of offspring birth weight, where the magnitude of association was the same in both groups, is attributable to prenatal risk effects.

It has been widely assumed that it is possible to deal with the contribution of inherited factors and other confounds by including variables such as parent ADHD in analyses and statistically controlling for confounding factors in large observational studies. Our results suggest that this might not be a safe assumption. Using a genetically sensitive “natural experiment” design provides a different way of testing the prenatal environmental mediation hypothesis, because it allowed us experimentally to remove inherited confounds and not rely on statistical controls that in this instance, for ADHD, are not adequate.

Community-based clinical studies and pooled analysis (where mothers and offspring are related) have consistently shown that exposure to smoking in pregnancy is associated with ADHD (1,3,4). Many studies show a dose–response relationship with the number of cigarettes smoked during pregnancy, and there are biologically plausible mechanisms that could explain this link (11,23). Thus, smoking in pregnancy has come to be viewed as an important environmental risk factor for ADHD. However, when the results of our study are considered together with other emerging findings, there are several reasons to be cautious in assuming smoking plays a causal role in ADHD.

First, in contrast to studies of birth weight, animal studies on cigarette and nicotine exposure in utero have not consistently demonstrated motor and cognitive changes similar to ADHD (11,12,14). Second, studies examining the children of twins reveal environmentally mediated effects of maternal smoking in pregnancy on birth weight (24) but not ADHD, at least in the offspring of alcoholics (25). Finally, studies examining siblings discordant for exposure to smoking in pregnancy suggest that maternal smoking in pregnancy might be indexing unmeasured familial risk that is not tapped by measurement of the usual confounders. Siblings not exposed to smoking in pregnancy but from families where mother smoked in another pregnancy show increased attentional, behavioral, and scholastic problems (26,27). Thus, when results from all these designs are considered together, a consistent pattern of findings emerges that suggest that the association between maternal smoking in pregnancy and offspring ADHD, in contrast to offspring birth weight, might not be causal (28). The shared inherited liability between maternal smoking in pregnancy and offspring ADHD symptoms also raises the possibility that there might be genetic risk variants that confer susceptibility to both nicotine dependence and ADHD.

How important are natural experimental designs such as ours when much larger observational studies are available? Epidemiological studies suggest that measured confounders do not account for the link between smoking in pregnancy and offspring ADHD (3,4), and in accordance with this, association in our related group remained when including covariates. That is, the inherited effect does not seem to be indexed by measured confounders and so would not be picked up by large observational studies.

Interestingly mother's current smoking and the father's smoking in pregnancy were also associated with offspring ADHD in the maternally related and paternally related groups, respectively, and the degree of association for current smoking and paternal smoking in pregnancy was similar to that for maternal smoking in pregnancy. These observations also further indicate that this maternal (and paternal) behavior is indexing inherited liability for ADHD. If paternal smoking was important in terms of effects through passive smoking effects on the fetus, we would have expected to also observe association in the paternally unrelated sperm donation and embryo donation groups but not the related surrogacy group, because surrogates do not live with father; but we do not observe this (results available from first author).

In terms of potential clinical implications, our findings together with those from other studies suggest that it is important for mothers to quit smoking in pregnancy and that smoking cessation could result in increased offspring birth weight but that this intervention might not be a useful public health intervention for reducing ADHD symptoms.

As with all research designs, there are limitations. First, although with this sample size we were able to detect association between maternal smoking in pregnancy and birth weight in the unrelated group, we cannot rule out that association was not detected for ADHD, given the small number of smokers in our sample and in the unrelated group in particular. As a result, our power to detect significant prenatal environmental influences was low, and future replications are needed. Thus, we cannot be certain that there are no prenatal effects, only that there is significant evidence of inherited effects. Notwithstanding this observation, our findings, at the very least, suggest that a proportion of the association between smoking in pregnancy and ADHD observed in previous studies can be attributed to inherited factors. Second, we did not examine ADHD diagnoses; however, all the research to date suggests etiologic continuity between normal variation in ADHD symptoms and extreme scores (29,30). Third, families that have used Assisted Reproductive Technologies might be unusual. However, comparison with representative community samples showed that family income and measures of psychological adjustment are similar (17). The income distribution in this sample was expected, given that fertility treatment is freely available in the United Kingdom under the National Health Service.

Unsurprisingly, the rate of maternal smoking in this sample was lower than currently reported UK rates. It is likely therefore that the mothers who smoked in our sample are the most highly dependent on nicotine, which could potentially increase the shared genetic liability with ADHD. However, the magnitude of association between maternal smoking in pregnancy and ADHD in the related group is similar to that reported in general population samples (19). Another potential issue is that there are some differences between the related and unrelated groups. Given that every design has its own set of strengths and weaknesses, different designs are needed, and there can be greater confidence when there are similar findings across a variety of designs, as is the case here (28).

### Conclusions

Our findings highlight the need to test causal hypotheses with genetically sensitive designs (7). Results from traditional observational designs do not necessarily pick up inherited confounds and could therefore be misleading. Our results suggest that the previously observed association between maternal smoking in pregnancy and ADHD might represent an inherited confound.

## Figures and Tables

**Table 1:** Description of Sample—Numbers of Children Related to Mother and Father

	Related Father	Unrelated Father
Related Mother	395	189
Unrelated Mother	172	36

Children (*n* = 23) from the gestational surrogate group are unrelated to the woman who undergoes the pregnancy but are related to the parents.

**Table 2:** Description of Sample

	Mother Related to Offspring	Mother Unrelated to Offspring	Statistical Test	*p*
Family Income Group in US $	$60,000–$80,000	$60,000–$80,000	Kendall τ = .08	.04
Mother Age at Pregnancy (Mean ± SD)	34.06 ± 3.63	38.72 ± 5.96	*t* = 13.52	.001
Prenatal Smoking (%)	4.9	6.5	χ^2^ = .753	.385
Prenatal Alcohol Use (Yes/No)	25.0	20.4	χ^2^ = 1.85	.173
Maternal ADHD Scores	8.25 ± 5.86	8.68 ± 5.85	.919	.358
Paternal ADHD Scores	8.36 ± 6.33	8.30 ± 6.33	*t* = −.088	.930
Singleton Births (%)	77.6	75.2	χ^2^ = .514	.474
Child Age, yrs (Mean ± SD)	6.82 ± 1.27	6.47 ± 1.33	*t* = −3.56	.001
Child Gender (% Male)	51%	49%	χ^2^ = .928	.335
Child Birth Weight, g (Mean ± SD)	3083.14 ± 677.08	3039.07 ± 755.49	*t* = −.811	.418
Child ADHD symptoms (Mean ± SD)	10.20 ± 7.30	11.58 ± 7.54	*t* = 2.33	.02

ADHD, attention-deficit/hyperactivity disorder.

**Table 3:** Results of Multiple Regression Analysis Testing for Association Between Exposure to Maternal Smoking in Pregnancy and Offspring ADHD Scores, Including Covariates in the Related Group

	β Unstandardized Coefficient	SE	β Standardized Coefficient	*p*
Smoking in Pregnancy	3.131	1.498	.103	.037
Child Gender (M = 1, F = 2)	−1.592	.731	−.107	.030
Child Age	−.288	.291	−.049	.324
Multiple Birth (Yes = 1, No = 2)	−1.655	.091	−.091	.064
Maternal ADHD	.363	.062	.294	<.001
Paternal ADHD	.195	.059	.165	.001
Family Income	−.305	.209	−.072	.146

ADHD, attention-deficit/hyperactivity disorder.
